# Evaluation of serum Vitamin-D levels in non-specific chronic musculoskeletal pain

**DOI:** 10.6026/973206300212740

**Published:** 2025-08-31

**Authors:** Pragati Khanorkar, Sangeeta Gupta, Parth Jani, Arun Deepak, Amrit Podder

**Affiliations:** 1 Department of Biochemistry, Datta Meghe Medical College, Nagpur, Maharashtra, India; 2 Department of Physiology, AIIMS Gorakhpur, Uttar Pradesh, India; 3 Department of General Medicine, AIIMS, Rajkot, India; 4 Department of Orthodontics, RVS Dental College and Hospital, The Tamil Nadu Dr. M.G.R. Medical University, Coimbatore, Tamil Nadu, India; 5 Department of Physiology, Teerthanker Mahaveer Medical College & Research Centre, Teerthanker Mahaveer University, Moradabad, Uttar Pradesh, India

**Keywords:** Vitamin D deficiency, musculoskeletal pain, chronic pain, 25-hydroxyvitamin D, non-specific pain, pain severity

## Abstract

Chronic non-specific musculoskeletal pain (CNMP) is a common condition, with recent studies suggesting a link to vitamin D deficiency. Therefore, it is of interest to measure serum vitamin D levels in CNMP patients and assesses its relationship to pain severity. Conducted over six months at a tertiary care center, 120 patients were enrolled and assessed for serum vitamin D levels and pain using the Visual Analog Scale (VAS). Results revealed 70% of patients were vitamin D deficient, with lower levels correlating with higher pain scores. Thus, we show that addressing vitamin D deficiency may be a cost-effective approach to managing CNMP.

## Background:

Chronic non-specific musculoskeletal pain (CNMP) is a common issue that affects a large number of people, often without a clear cause [1]. Recent studies indicate that a lack of vitamin D might contribute to the development and ongoing nature of CNMP [2]. Vitamin D, which is a type of secosteroid hormone, is crucial not just for maintaining healthy bones but also for muscle function and managing inflammation [3]. Research has shown that many individuals with chronic musculoskeletal pain have low levels of vitamin D. Studies in India found a significant number of patients with chronic low back pain were vitamin D deficient [4]. Intervention studies have also pointed out the potential benefits of vitamin D supplementation for easing musculoskeletal pain [5]. Few researchers noted that patients with CNMP experienced significant relief from pain after taking vitamin D and calcium supplements and found that vitamin D replacement therapy led to considerable reductions in pain severity for those who were deficient [6]. However, even with the increasing interest in how vitamin D levels relate to musculoskeletal pain, the evidence is still mixed, highlighting the need for more research across different populations [7, 8]. Therefore, it is of interest to evaluate serum vitamin D levels in patients with chronic non-specific musculoskeletal pain and to investigate the potential implications for clinical treatment.

## Methodology:

This study was a cross-sectional observational research project carried out over six months at a tertiary care hospital. The goal was to assess serum vitamin D levels in individuals suffering from chronic, non-specific musculoskeletal pain. Before starting the study, we secured ethical approval from the institutional ethics committee, and all participants gave their informed written consent. We included adult patients aged 18 to 65 who had been experiencing musculoskeletal pain for over three months without any identifiable underlying issues like trauma, inflammatory arthritis, cancer, or systemic illnesses. To avoid any interference with serum vitamin D levels, we excluded patients who had taken vitamin D supplements in the last three months, as well as those with known metabolic bone disorders, kidney or liver problems, or thyroid dysfunction. Each participant went through a thorough clinical evaluation, which involved a detailed history of their pain characteristics, duration, and any related symptoms. We conducted a comprehensive physical examination to rule out any specific musculoskeletal or neurological issues. We also gathered relevant demographic and clinical information, such as age, gender, occupation, sunlight exposure, and dietary habits, using a structured questionnaire. Fasting venous blood samples were taken from all participants to measure serum 25-hydroxyvitamin D [25(OH)D] levels through a chemiluminescent immunoassay technique. We classified vitamin D status as deficient (<20 ng/mL), insufficient (20-29 ng/mL), or sufficient (≥30 ng/mL) according to Endocrine Society guidelines. Additionally, we performed other laboratory tests, including serum calcium, phosphorus, alkaline phosphatase, thyroid function tests, and renal function tests, to rule out other possible causes of musculoskeletal pain. We gathered all the data and ran it through some statistical software for analysis. To give a clear picture of the demographic and clinical traits of our study group, we used descriptive statistics. For continuous variables, we calculated the mean and standard deviation, while categorical variables were shown as frequencies and percentages. We also looked into how vitamin D levels related to the clinical features of pain, using the right statistical tests, considered a p-value of less than 0.05 to be statistically significant.

## Results:

In a study involving 120 patients suffering from chronic non-specific musculoskeletal pain, the average age of participants was 42.7 years, with a standard deviation of 10.6 years. Notably, there was a higher representation of females, making up 61.7% of the group. Most of the patients, about 68.3%, came from urban settings and reported having limited sun exposure during their daily activities. The pain was primarily concentrated in the lower back, affecting 43.3% of the participants, while 30.8% experienced generalized body aches. When it comes to Vitamin D levels among those in the study, 84 patients, or 70%, were found to be vitamin D deficient (with levels below 20 ng/ mL). Additionally, 23 patients (19.2%) had insufficient levels (ranging from 20 to 29 ng/mL), and only 13 patients (10.8%) had sufficient levels (30 ng/mL or higher). The average serum 25 (OH) D level across all participants was 16.4 ng/mL, which highlights a significant prevalence of hypovitaminosis D in this group. There was a notable link between lower vitamin D levels and both the duration and intensity of musculoskeletal pain, with a p-value of less than 0.01.

Looking at the relationship between vitamin D levels and clinical characteristics, patients with vitamin D deficiency reported much higher pain scores on the Visual Analog Scale (VAS), averaging 7.8 compared to 4.6 for those with adequate vitamin D levels. Furthermore, the group lacking sufficient vitamin D also experienced more frequent fatigue and functional limitations. These findings reveal a significant inverse relationship between serum vitamin D levels and the intensity of musculoskeletal pain. The high rate of deficiency among patients indicates that low vitamin D levels might be an overlooked factor contributing to chronic musculoskeletal discomfort in this group. Table 1 (see PDF) presents the demographic and clinical profile of the study participants, including their mean age, gender distribution, residence type, sunlight exposure, and common sites of pain. The majority of participants were female (61.7%) and resided in urban areas (68.3%), with a significant number reporting limited sunlight exposure (75.8%). Common sites of pain included the lower back (43.3%), generalized body pain (30.8%), and shoulder/neck pain (15%). Table 2 (see PDF) displays the distribution of participants by Vitamin D status and their corresponding pain scores. It shows that most participants had deficient Vitamin D levels (<20 ng/mL) with a mean serum level of 12.1 ng/mL and a high mean VAS pain score of 7.8. Those with insufficient (20-29 ng/mL) and sufficient (≥30 ng/mL) levels of Vitamin D had lower pain scores. Figure 1 (see PDF) illustrates the distribution of Vitamin D status among the study participants, emphasizing the higher prevalence of Vitamin D deficiency. Figure 2 (see PDF) shows the correlation between serum Vitamin D levels and VAS pain scores, highlighting a negative correlation, where lower Vitamin D levels correspond to higher pain intensity.

## Discussion:

The current study revealed a strikingly high rate of vitamin D deficiency among people suffering from chronic, non-specific musculoskeletal pain, with a staggering 70% of participants showing serum 25 (OH) D levels below 20 ng/mL. Additionally, we found a significant negative correlation between serum vitamin D levels and pain severity, as assessed by the Visual Analog Scale. These results bolster the growing evidence that low vitamin D levels may play a role in the development of unexplained chronic musculoskeletal pain. Prakash *et al.* (2013) [9] investigated the relationship between chronic musculoskeletal pain, tension-type headaches, and vitamin D deficiency, proposing that subclinical osteomalacia could be a common underlying factor for both symptom sets. Their findings emphasize the wider systemic effects of vitamin D deficiency, which resonate with our observation that functional limitations and fatigue were more pronounced in those with low vitamin D levels. This shared symptomatology highlights the importance for healthcare providers to consider vitamin D testing in patients presenting with overlapping non-specific pain issues. Similar findings have emerged in earlier research involving various populations. For instance, a study in Ethiopia by Zenebe *et al.* (2020) [10] found that most patients experiencing generalized neuromuscular pain and fatigue had low vitamin D levels, echoing what we observed in our group. Knutsen *et al.* (2010) [11] also reported a high occurrence of vitamin D insufficiency among patients with musculoskeletal pain, fatigue, and headaches in a diverse general practice in Norway, suggesting that this connection might be relevant worldwide, regardless of ethnicity or geographic location.

Abdul-Razzak and Kofahi (2020) [12] highlighted the importance of vitamin D for musculoskeletal health by linking low vitamin D levels to carpal tunnel syndrome, suggesting that a deficiency could hinder neuromuscular function. While our current study didn't specifically target neuropathic pain syndromes, the functional effects observed in our patients lend support to a similar understanding. On the other hand, research by Bjelakovic *et al.* (2021) [13] and Rahimpour *et al.* (2022) [14] has leaned more towards exploring vitamin D's impact on liver health and its metabolic advantages. However, it's crucial to remember that vitamin D acts as a regulatory hormone with significant roles in muscle metabolism and inflammation. Moreover, recent studies, such as those by Alonso-Pérez *et al.* (2024) [15] and Chaudhari *et al.* (2023) [16], provide additional evidence of the role of vitamin D in pain management. Alonso-Pérez *et al.* highlighted the potential benefits of vitamin D supplementation in reducing pain and inflammation, while Chaudhari *et al.* emphasized the relationship between vitamin D status and overall well-being in individuals suffering from chronic conditions. These findings further support the notion that addressing vitamin D deficiency could contribute to alleviating the symptoms of musculoskeletal pain, improving patient outcomes. These broader implications strengthen the case for vitamin D screening not just in liver disease but also in chronic pain syndromes, especially when other potential causes have been excluded. In summary, our findings back the idea that vitamin D deficiency is both common and clinically significant for patients dealing with chronic non-specific musculoskeletal pain. Considering how simple and affordable vitamin D testing and supplementation are, implementing routine screenings for this patient group could provide substantial clinical benefits, particularly in areas where deficiency rates are high. More longitudinal studies are needed to determine if consistently correcting vitamin D levels leads to lasting pain relief and improved function.

## Conclusion:

A strong link between vitamin D deficiency and chronic non-specific musculoskeletal pain is shown. Low serum 25(OH) D levels are associated with higher pain severity, suggesting vitamin D deficiency may play a key role in these pain syndromes. Regular screening and addressing vitamin D deficiency could be a cost-effective strategy for managing unexplained musculoskeletal pain, though further studies are needed to confirm long-term benefits.
